# The effects of aggressive environments on the mechanical properties of basalt plastics

**DOI:** 10.1016/j.heliyon.2020.e03481

**Published:** 2020-03-04

**Authors:** M.P. Lebedev, O.V. Startsev, A.K. Kychkin

**Affiliations:** Federal Research Center "Yakut Scientific Center of the Siberian Branch of the Russian Academy of Sciences", V.P. Larionov Institute of Physical and Technical Problems of the North, Yakutsk, Russian Federation

**Keywords:** Materials science, Basalt fiber, Basalt plastic, Chemical composition, Alkali resistance, Strength, Young's modulus, Mechanical indicators, Climatic tests

## Abstract

A review and analysis of changes in mechanical properties of basalt fibers (BF) and basalt composite materials under effects of aggressive environments in reference to foreign and Russian scientific literature has been carried out. The changes in the physical and mechanical properties of the fiberglass (FG) and basalt plastic (BP) have been compared. The analysis shows that BF is a good alternative to fiberglass for elaboration of composite materials for various purposes. In the most aggressive alkaline environments, BP resistance is higher as compared with that of FG. If required, BF resistance to chemically active media could be increased by midifying its composition and applying protective layers and heat treatment of fibers. For extended use of BP as an effective structural material under various climatic conditions, including in the Arctic, long-term tests of 10 or more years are advisable and relevant, with control of changes in their mechanical parameters and an analysis of processes of climatic aging developing in BP.

## 1 Introduction

Over the past two decades, application of basalt fibers (BF), as an alternative to glass fibers (GF), has been expanding rapidly [1, 2, 3, 4, 5, 6, 7, 8, 9, 10]. BF is obtained from various rocks containing an eight-component system of oxides Na_2_O-K_2_O-MgO-CaO-FeO-Fe_2_O_3_-SiO_2_-Al_2_O_3_, with a small content of titanium oxide (up to 1 wt.%) [9, 10]. The major purpose of BF is related to development of basalt plastics (BP), which reinforcing elements are strong and rigid bundles of fibers, threads, fabrics, nets for manufacture of a large range of various cheap structural elements: (rods for concrete reinforcement [1, 2], road structures [3], pipes [4], bridge elements [4, 5], details of transport products, sports equipment, wind turbines [6], offshore structures [7], etc.).

Basalts from Yakutia's deposits make a promising material for production of BF and manufacture of building fittings. The aim of our study is substantiation of widespread application of BP for construction of various buildings and structures in cold climates. To this end, an analysis of results of recent scientific and technical research has been conducted.

## 2 Comparison of properties of basalt fibers with those of other fibers

A series of informative reviews are presented in the scientific and technical literature, in which effects of the chemical composition of BF on their mechanical properties are analyzed, and a comparison is made with properties of GF. For example, in [3, 8, 9, 10], the content of oxides in BF is compared with the three types of GF (E-glass — aluminoborosilicate glasses with less alkaline elements and with replacement of sodium and potassium by boron oxide, S-glass — aluminosilicate glass with a high magnesium content of increased strength, ZrO_2_-glass is chemically resistant glass containing zirconium oxide). The presence of Fe_2_O_3_ is the reason for the gray-brown color of BF. Table 1 shows the chemical composition of BF and GF.

**Table 1:** Chemical composition of BF and GF[3, 8, 9, 10].

Major components	Composition∗, mass %		
	BF	E-glass	S-glass
SiO_2_	48.8–57.5 47.5–55	52–56 52–60	64–66 60–65
Al_2_O_3_	14–18.2 14–20	12–16 12–15	24–26 23–35
CaO	5.2–10 7–11	16–25 21–23	0–0.3 0–9
MgO	1.3–16 3–8.5	0–5 0.4–4	9–11 6–11
Na_2_O	1.9–6.4 2.5–7.5	0–2 0–1	0–0.3 0–0.1
K_2_O	0.8–4.5 2.5–7.5	0.2–0.8 0–1	- -
Fe_2_O_3_ +FeO	4.0–13.3 7.3–13	<0.3 0–0.4	<0.3 -

∗ The first line shows data [3, 8], the second line gives data [9, 10].

Variations in the chemical composition, fiber forming conditions (extract temperature, melting time), and the fiber diameter significantly affect the ultimate tensile strength σ_t_, Tensill strength E, and relative elongation in tension (Table 2) [1,3,6,8,[11], [12], [13], [14], [15], [16], [17], [18], [19], [20], [21], [22], [23]]. The mechanical characteristics of BF and GF are evident to be comparable, and in some cases, BF indicators have obvious advantages, as compared with the corresponding GF indicators. The levels of σ_t_ and E have various combinations. For example, for BF with E = 90 ± 3 GPa, the ultimate tensile strength σ_t_ varies from 2100 MPa [15] to 2800 MPa [20], 2895 MPa [12] and even 4840 MPa [16].

**Table 2:** Comparison of mechanical properties of BF and GF

Fiber type	Ultimate tensile strength, MPa	Tensill strength, GPa	Relative elongation in tension, %	Density, g/cm^3^	Fiber diameter, μm	Source
BF	1900–2600	70–90	3.5–4.5	2.6–2.8		1
E-glass	2800–3000	74–95	4.7–5.6	2.4–2.5		1
BF	3000–4840	93–110	3.1–6	2.63–2.8	6–21	3
E-glass	3100–3800	73–76	4.7	2.54–2.57	6–21	3
S-glass	4020–4650	83–97	5.3	2.54	6–21	3
BF	730–3470	35–103			6–21	6
E-glass	1035–1649	57–71			10–17	6
BF	4840	89	3.2	2.7		8
E-glass	3450	77	4.7	2.57		8
S-glass	4710	89	5.6	2.48		8
BF	1644		1.2	2.64	17	11
BF	2825	89		2.8		12
BF		85–95			10–22	13
E-glass		78				13
BF		84	2.8	2.67	13	14
BF	2100	91	2.6	2.8	11	15
E-glass	1500	80	1.9	2.6	12	15
BF	4840	89	3.1			16
E-glass	3450	73.4	4.7			16
S-glass	4580	85.5	5.6		9–22	16
BF	1060–1910	77–92				17
E-glass	1600	71.4			13	17
S-glass	1910	89.5			10	17
ZrO_2_-glass	1180	70			12	17
BF	4840	89	3.2	2.7		18
BF	1590	64	2.5	2.6	12	19
E-glass	1040	69	1.5	2.6	10	19
BF	2800	89	3.2	2.8		20
E-glass	1400–2500	76	1.8–3.2	2.56		20
BF	3000–4840	79–93	3.2	2.65–3.05		21
E-glass	3100–3800	76–81	4.6–4.7	2.55–2.62		21
S-glass	4600–4800	88–91	5.6	2.46–2.49		21
BF	3000–4800	79–93	3.1		6–21	22
E-glass	3100–3800	73–76	4.7		6–21	22
BF	1830	72.2	3.0	2.63	10.2	23
S-glass	2295	78.3	3.4	2.52	12.3	23

The chemical composition or molding conditions can be chosen from a large set of various glass options in order to achieve the required level of mechanical properties of BF, but in practice, the primary importance is given to stability of fibers in GF or BF for a long-term operation under aggressive conditions.

## 3 Resistance of BF and BP to temperatures and thermal cycles

According to some researchers [20, 24, 25, 26, 27, 28, 29, 30], BF and composites based on them are characterized by high resistance to high temperatures and thermal cycles. Comparative results of the conducted research are given in Table 3.

**Table 3:** Comparison of thermal stability of BF, GF and BP.

Fiber type	Thermal processing rates		Measured rate of fiber R			Source
	Temperature, ^о^С	Duration, hr	Rate	Initial value	Relative value of R after thermal processing ∗	
BF	100	2	Tensill strength, GPa	992	1.03	24
S-glass				1798	0.93	
BF	200			992	1.08	
S-glass				1798	0.94	
BF	400			992	0.97	
S-glass				1798	0.70	
BF	600			992	0.93	
S-glass				1798	0.55	
BF	500	4	Pore volume, m^3^/kg ∙ 10^−5^	4,6	0.31	25
	700				0.29	
	900				0.28	
	1000				0.26	
BF	200	2	Tensill modulus (numerator) and tensill strength (denumerator), GPa	80/2,3	0.98/0.96	26
E-glass				75/2.0	0.96/0.90	
BF	400			80/2,3	1.0/0.43	
E-glass				75/2.0	0.90/0.80	
BF	600			80/2,3	1.0/0.17	
E-glass				75/2.0	1.1/0.40	
BF	135	4		80.5/2.44	1.08/0.98	27
	300				1.01/0.67	
BP-T5 ∗∗	thermal cycles from –30 °C to +220 °C	duration of each cycle was 6 min		10.8/0.253	0.99/0.99	28
BP-T10 ∗∗					0.94/0.99	
BP-T15 ∗∗					0.94/0.95	
BP-T20 ∗∗					0.93/0/95	
BP-T25 ∗∗					0.93/0/95	
BP-T30 ∗∗					0.92/0.95	
BP	150	10		10.85/0.253	0.99/0.94	29
	200				0.97/0.90	
	250				0.93/0.82	
BP-T100 ∗∗	thermal cycles from –30 °C to +220 °C	duration of cycle was 12 h	Bond stress in concrete, MPa	18.1	0.89	30
BP-T200 ∗∗					0.91	

∗ is given the value R_T_/R_0_, where R_0_ – value in the initial state, R_T_ – value after thermal processing; ∗∗ the number in specimen codes indicate the number of thermal cycles.

To check the thermal stability in [24], BF and GF (S-glass) have been heated for 2 h at 100, 200, 400, 600, 1200 ^о^С. The ultimate tensile strength is measured a day after cooling. Upon heating to 200 ^о^С, the GF σ_t_ decreases by 6%, while in BF it increased by 8%. After heating to 600 °C, the BF strength index decreases by 7%, while in GF it decreases by 45%.

The authors in [24] believe that heat treatment improves the crystal structure of biologically active BF. The ability to crystallize depends on the chemical composition of basalt and the heating temperature [20]. Due to the presence of iron oxides, crystallization starts with oxidization of iron cations and formation of the spinel structural phase, which produces the densest cubic packing of oxygen anions. The bivalent cations Ca^2+^, Mg^2+^, Fe^2+^ diffuse from the inner volume to the surface, where they react with oxygen from the external medium, forming nanocrystalline layers of CaO, MgO, (Mg,Fe)_3_ O_4._

The high thermal stability of BF is proved in [25, 26]. Heat treatment of BF at 500–1000 ^о^С is accompanied by crystallization processes of fiber [25] and has positive effects on their density and thermo-physical properties. Upon heating to 600 ^о^С, the Tensill strength of BF remains unaltered at the level of 80 GPa [26], while the σ_t_ decreased by 60%. According to the data in [27], crystallization and structural rearrangements in BF upon exposure to 135 and 300 ^о^С also do not reduce the E index, but they reduce the value of the tensile strength from 2440 MPa to 1640 MPa. Thus, the influence of the chemical composition of basalt on the thermal stability of BF requires further study.

In [28], the mechanical indices of a 4-layer BP based on a phenolic binder are monitored during thermal cycling from -30 to 220 °C. After 30 exposure cycles, the values of σ_t_ and E decrease only by 5–7%. In a similar study [29], BP indices based on a phenolic binder decrease by 7–18% after 10 h of heating at 150, 200, and 250 ^о^С. The data given in [30] also show that BP is not inferior in terms of thermal stability to GP.

## 4 Resistance of BF to chemically active environments

### 4.1 Water and aqueous solutions of salts

Comparative results of the study of water effects on BF and BP properties are given in Table 4.

**Table 4:** Results of study of water effects on BF and BP properties.

Material	Effect modes		Measured rate, R			Source
	Temperature, ^о^С	Duration, hr	Rate	Initial value	After water effects	
BF	100	3	Weight loss after boiling, % wt	0	1.6	3
E-glass					6.2	
S-glass					5.0	
BP	40 in normal water	5760	Short Beam Strength, MPa	54	30	8
GP				20	20	
BP	40 in sea water			54	28	
GP				20	20	
BF	96	168	Weight loss after exposure to H_2_O, % wt	0	0.2	13
			Weight loss after exposure to aqueous solution of NaCl, % wt	0	0.3	
BF	100	3	Tensile strength, MPa	1828	1824	23
BF _43% SiO2_	70	672	DSC peak temperature, ^о^С	894	906	31
BF _49% SiO2_				893	902	
BF	Room temperature	120	Normal water absorption, %wt	0	0.3	32
			Sea water absorption, %wt		0.45	
BP			Flexural strength after normal water absorption, MPa	461	454	
			Flexural strength after sea water absorption, MPa		447	
BF	100	3	Weight loss after boiling, % wt	0	1.0	33
BP	40	4800	Flexural modulus, GPa	38.4	36.5	34
GP				38.0	36.4	
BP			Flexural strength, MPa	698	526	
GP				594	559	
BP	80	2400	Normal water absorption, %	0	4.0	35
GP					6.0	
BP			Tensile strength, MPa	440	340	
GP				285	120	
BP			Short beam strength, MPa	25	13	
GP				16	8	

Review [3] shows the advantages of BF as compared to GF, based on E-glass and S-glass for lower weight loss after 3 h of boiling in water. The authors of [31] study two types of BF with the 43 and 49% of SiO_2_ content. After 4 weeks in water at 70 ^о^С, leaching of elements occurs modifying the lattice. Chemical aging affects 50–200 nm of the surface layer of BF and contributes to its crystallization. Crystallization is confirmed by the shift of the exothermic peak to 9–12 ^о^С and higher on the DSC curve. Losses of BF mass after 7 days in distilled water and aqueous NaCl at 96 ^о^С are 0.2–0.3% [13]. After 3 h boiling of BF in water, its σ_t_ index do not change [23]. According to the data in [32], BF increases its mass after 5 days of immersion in distilled and sea water by 0.3 and 0.45%, respectively. In [33], ropes made from BF have been boiled in water for 3 h and, after drying, the 1% mass loss is detected. Since alkaline R ions are more mobile in the volume of basalt glass, a reaction characteristic of water occurs on the surface which does not significantly affect the strength of the rope.

(1) ≡Si–OR + 2(H^+^ + OH^–^) → ≡Si + OH + ROH

Effects of water and aqueous solutions of salts on BF properties have been studied in [8, 32, 34, 35]. BF and GF based on an epoxy binder, have been aged in sea water at 40 ^о^С for 200 days [34], with the same level of water absorption (1.3–1.5%), shows that the Tensill strength in bending remains at the level of 95–96%, and evidences the same decrease in tensile strength at the interlayer shear (19–22%), but different values of the tensile strength in bending (75% and 95%). The cyclic 4-point bending has been studied at 2 Hz and the cycle asymmetry coefficient R = 0.1 and differences in the course of fatigue curves for samples previously aged in sea water and samples without moisture saturation have been shown. The drop of the fatigue strength of the GP is less, but the initial BP indicators are better. Report [8] examines aging of BP and GP in the normal and salt water at 40 ^о^С for 240 days, exposure to moisture, temperature, mechanical fatigue loading, and 199 cycles of freezing and thawing. The filler-matrix interface in BP has been shown as more sensitive to impacts than that in GF. However, it has not been shown how to correlate the effects of damage to the interface with the effects of deterioration in BF values.

In [35], properties of BP and GP based on epoxy binder and fibers of the same thickness have been compared. The samples have been held in water at 80 ^о^С for more than 100 days. GP absorbs about 6% of water, while BP absorbs about 4 % of water. The rate of water saturation of BP is lower than that of GP. The effects of a decrease in the BP strength with water saturation are less than in GP: BP σ_t_ decreases by 36% (GP σ_t_ decreases by 67%), the short beam strength of BP decreases by 26% (that of GP decreased by 59%). It has been concluded in [35], that the interaction between the epoxy matrix and BF is higher than between this matrix and GF.

Thus, when considering the effect of water and aqueous salt solutions on BP properties, one can assume that the water resistance of this class of composite materials is not inferior to that of GP, and in some cases it is even higher. We can hypothesize that with a high BF stability, the main reasons for the decrease in BP mechanical properties are physical and chemical transformations in polymer matrices and at the polymer-filler interface. However, the reversible effects of moisture plasticization and the irreversible destruction effects, previously analyzed in detail for GP [36, 37, 38, 39, 40, 41], are not discussed in the reviewed works and should be investigated additionally. To do this, it is advisable to use informative and well-established methods of the dynamic mechanical analysis, thermomechanical analysis, linear dilatometry, calorimetry, and to reveal the mechanisms of physical and chemical transformations in the volume and on the surface of the samples [41].

### 4.2 Acid solutions

Comparative results of study of effects of acid solutions on BF and BP properties are shown in Table 5.

**Table 5:** Results of study of effects of acid solutions on BF and BP properties.

Material	Effect modes		Measures rate R			Source
	Temperature, ^о^С	Duration, hr	Rate	Initial value	After effect of acid	
BF	96	168	Weight loss after aging in H_2_O, % wt	0	0.2	13
			Weight loss after exposure to aqueous solution NaCl, % wt		0.3	
			Weight loss after exposure to alkali solution, % wt		0.9	
			Weight loss after exposure to acid solution, % wt		5.9	
BF	100	3	Weight loss after boiling in water, % wt	0	1.0	33
			Weight loss after boiling in NaOH solution, % wt		4.0	
			Weight loss after boiling in HCl solution, % wt		5.0	
			Breaking force after boiling in HCl solution, kN	3.16	2.44	
BP	100	3	Tensile strength after acid treated fibers, MPa	1220	1150	42
GP				1100	500	
BP	Room temperature	24	Tensill strength after acid treated fibers, MPa	198	246	43
GP				180	198	
BP			Shear strength after acid treated fibers, MPa	20.6	21.1	
GP				15.6	16.3	
BP			Impact strength after acid treated fibers, J/mm	2.42	2.86	
GP				1.48	1.69	
BP	55	1584	Tensile strength after exposure in acid solution, MPa	1908	1636	44
GP				1291	1206	
BP			Tensile modulus after exposure in acid solution, GPa	77.1	77.7	
GP				73.7	77.3	

Acid solutions cause the highest weight loss of BF as compared with other chemically active environments. For example in [13], the weight loss of BF after 7 days in water at 96 ^о^С is 0.3%, in saline medium 0.2%, in alkali 0.9%, and in acid 5.9%. The properties of BF have beene studied after 3 h of boiling in a 2 - molar aqueous solution of hydrochloric acid. The mass of the samples after boiling in water decreases by only 0.4%, which corresponds to the data of [13, 32], while the mass loss in the HCl solution reaches 8.1%. The ultimate tensile strength of fibers after exposure to moisture do not change, and after exposure to the acid solution it decreased by 34%. After boiling in acid, the mass amount of Si on the surface of the BF increases from 31% to 69%, while the content of metal elements Na, Mg, Al, K, Ca, Fe increases (a total of 39 wt.%). It has been concluded that metal atoms change places with H + in acid. Due to this, the lattice structure on the fiber surface is destroyed, resulting to a decrease in strength.

According to [33], a 3-hour boiling of BF ropes in a 2-molar aqueous solution of hydrochloric acid causes a 5% decrease in the mass of samples. Microphotographs of BF show the smooth surfaces of fibers soaked in water and acid. An elemental analysis reveals an increase in the Na content by 0.7%, Al by 1.6%, Fe by 1.5%, Mg by 0.63%. Thus, it has been proved that the structure of silicon oxide is resistant to acids, and metal ions change places with hydrogen atoms and pass into an acid solution in accordance with the reaction [42].

(2) ≡Si–OM + H^–^ → ≡Si–OH + M^+^.

In [43], BP and GP (E-glass) based on unsaturated polyester with untreated BF and GF (E-glass) and fibers previously stored in a H_2_SO_4_ solution have been studied. The BP advantage over GP has been revealed both without treatment, and after exposure to a solution of sulfuric acid in terms of the ultimate tensile strength, interlayer shear, and the Izod impact strength. The highest value of σt is achieved in BP after treatment of the fibers with acid. In BF, the reaction occurs under the action of acid [43].

(3) Fe_2_O_3_ + 3H_2_SO_4_ → Fe_2_(SO_4_)_3_ + 3H_2_O.

After treatment of BF in acid, two groups of bonds are created: covalent bonds between surface –OH groups of ester with carboxylic acid and hydrogen bonds between carbonyl groups of the ester and OH groups of the fiber, which positively affect the mechanical properties of BP [43]. Wherein, the preliminary chemical modification of BF reduces the water absorption of BP [32]. An example of a comparative assessment of effects of aqueous acid solutions on the properties of BP and GP is given in [44], which proves the need to control reversible and irreversible transformations in an epoxy matrix. The high resistance of BF and BP to effects of acid solutions are also noted by other authors, for example in [9, 10, 17, 21].

### 4.3 Alkali solutions

An increased attention is paid to the analysis of effects of alkalis on the properties of BF and BP in the scientific literature, since the use of reinforcing elements made from BP is most promising in concrete with a pronounced alkaline environment [1, 2, 7].

Alkaline solutions have the greatest impact on the structure and properties of BF [9, 10, 18, 23, 24, 27, 33, 42, 45].

Comparative results of study of effects of acid solutions on BF and BP properties are given in Table 6.

**Table 6:** Results of study of effects of alkali solutions on BF and BP properties.

Material	Effect modes		Measured rate R			Source
	Temperature, ^о^С	Duration, hr	Rate	Initial value	After effects of alkali	
BP	Room temperature	8760	Coefficients of long-term resistances after exposure in dry state	1,0	0.67	1
			Coefficients of long-term resistances after exposure in 1% NaOH		0.22	
BF	Room temperature	240	Tensile strength after exposure in alkaline solution, MPa	1262	1117	9
			Tensile modulus after exposure in alkaline solution, GPa	48	48	
BF	100	3	Weight loss after exposure to NaOH, % wt	0	15	10
BP	Room temperature	2400	Shear strength reduction at alkali aging	1.0	0.87	17
GP					0.47	
BF	100	1	Mass loss ratio after being boiled in alkaline solution, %	0	2.8	18
E-glass					3.7	
BF	100	3			4.3	23
BF	40	672	Tensile strength after NaOH solution treated fibers, MPa	992	200	24
S-glass				1798	270	
BF	20	98	Tensile strength after exposure in alkaline solution, MPa	2.44	1.0	27
	40				0.75	
	60				0.70	
	20	720	Tensile modulus after exposure in alkaline solution, MPa	80.5	62.8	
	40				58.7	
	60				55.6	
BP	60	504	Breaking stress after exposure in water, MPa	1070	1050	33
			Breaking stress after exposure in NaCl solution, MPa		1230	
			Breaking stress after exposure in alkaline solution, MPa		600	
BF	100	0.5	Tensill strength after exposure in alkaline solution, GPa	2600	600	45
E-glass				2500	330	
BP	60	8742	Creep Rupture Stress Limit Coefficient	1.0	0.35	46

It has been shown in [27, 33, 42], that the main result of alkali impacts on BF is destruction of the SiO_2_ lattice under the effects of hydroxyl groups in the surface layer causing leaching of potassium, aluminum, and sodium with an increase of the content of magnesium, iron, titanium, and calcium. Thus, the authors of [23] show an increase in the Na content from 4.5 to 10.8% on the surface of BF after their 3-hour boiling in NaOH solution.

(4) ≡Si–O–Si≡ + OH^–^ → ≡Si–OH + ≡Si–O^–^,

The BF volume decreases with formation of gel or surface corroded layers, and in an alkaline solution, the amount of K, Si Al [33] increases up to 2–4%, resulting in significant alterations of mechanical properties of fibers [24]. For example, after 2 weeks of BF exposure to alkaline solution, the σ_t_ index decreases from 2440 MPa to 1000 MPa at 20 °C, and to 750 MPa at 40 °C and 700 MPa at 60 °C [27]. Young's modulus also decreases from 80.5 to 63, 59, and 56 GPa, respectively. The relative elongation at break sharply decreases (from 3.1% to 0.8%).

According to the data of [9, 10], the resistance of BF to aggressive environments strongly depends on the content of metal oxides in fibers and their ratio. A parameter is proposed for prediction of the chemical resistance of fibers [10].

(5) $$N_{x}=\frac{SiO_{2}+Al_{2}O_{3}+Fe_{2}O_{3}}{CaO+MgO+K_{2}O+Na_{2}O},$$

considering interactions of SiO_2_, Al_2_O_3_, Fe_2_O_3_, CaO, MgO, Na_2_O, K_2_O. It has been shown that in assessing chemical resistance, a mass loss can only indirectly characterize the stability of fibers in aggressive environments. A more accurate assessment of the chemical resistance of BF is provided by the results of changes in their tensile strength after exposure to acids and alkalis. The N_x_ parameter determined by relation (5) takes into account the ratio of acidic and basic metal oxides in basalt glasses in mass. % [10]. An increase in the alkali resistance with an increase in the N_x_ parameter was experimentally proved by a change in σt [10]. The higher the N_x_ of the feedstock, the greater the stability of fibers in aggressive environments.

In survey [18], mechanical properties and chemical resistibility of BF has been analyzed. Alkalis are concluded to be more active than acids. The chemical resistance of BF is noted to depend on the temperature, fiber composition, duration of exposure, chemical composition of the agent, pH of the solution, and the fiber size. This conclusion is consistent with the results of work [45], in which the resistance of BF and GF (E-glass) has been compared at 3-hour exposure in boiling NaOH and HCl solutions with a concentration of 2 mol/L. The data on the chemical composition given above allows us to predict higher resistance of BF, since for them, the chemical stability parameter Nx, determined by relation (5), is 4.6, and for CB Nx = 2.9. After 3 h treatment in an acid solution, 10% of the mass of GF and 40% of the mass of BF are lost. While, 35% of σ_t_ of BF and only 10% of the similar value of GF are retained. Since the –Si – O– structure is inert to acid [42], after 3 h of treatment in HCl solution, the amount of Na, Mg, Al, K, Ca, Ni, and Fe on the surface of the BF decreases, while the content of Si increases. Microphotographs show a distinct layer of corrosion in BF. After 3 h of exposure in alkali, 2% of the mass of GF and 7% of the mass of BF are lost. Only 10% of GF σ_t_ is retained, and the value of this indicator in the BF drops to almost zero.

Despite such significant changes in the properties of BF in the reviewed test solutions, the alkali resistance of BP is considered to be quite satisfactory. For example, in simulation of stay in concrete, creep failure was studied for BP rods with a diameter of 4.3 mm at a load of 25–80% of the limiting initial level upon exposure to an alkaline solution and at 60 °C [46]. The predicted value of σt after 50 years is 18%, and after 114 years it makes 13%. Comparative tests of BP rods based on epoxy and vinyl ester matrices in a similar alkaline medium at 60 °C for 7 months show [13], that ultimate strength at interlayer shear decreases by 9 and 23%, with bending by 27–29%. Young's modulus decreases by 14–16%, and the water absorption of BP on the epoxy matrix is 1.4 times higher than that on the vinyl ether matrix. Thus, the assessment of the state of BP in alkaline media depends on the test conditions and the choice of polymer binders, which is confirmed by the results of [1, 17, 20, 47, 48, 49].

The resistance of BP to alkali can be increased if a protective layer containing ZrO_2_ is applied to the surface of BF. The more insoluble compounds Zr^4+^, Fe^3+^, Mg^2+^ are contained in the corrosion layer, the higher the alkali resistance [50]. Other ways to increase the resistance of BP to alkaline media of concrete are considered in review [51]. In particular, it is recommended to choose the optimal composition of binders based on vinyl ester resins, epoxy resins, thermoplastic polymer matrix with reduced porosity at the polymer-fiber interface, which increases shear resistance and freeze-thaw resistance.

## 5 Climatic stability of BF

Aging of BP under natural climatic conditions has not been studied well enough [1, 24, 52, 53, 54, 55, 56]. Some results of the study of effects of acid solutions on BF and BP properties are given in Table 7.

**Table 7:** Results of study of effects of climatic ageing on BP properties.

Material	Modes of effects		Measured rate R			Source
	Climate type	Duration, year	Rate	Initial value	After exposure	
BP	Warm wet	0.5	Tensile strength after exposure without stress, MPa	340	330	1
			Tensile strength after exposure with 0.2 stress, MPa		315	
			Tensile strength after exposure with 0.45 stress, MPa		302	
			Tensile strength after exposure with 0.7 stress, MPa		220	
BF	Artificial exposure	20	Tensile strength after NaOH solution treated fibers, MPa	1392	1207	24
S-glass				2182	1828	
BP	Warm wet	1	Tensile strength, MPa	78	66	52
			Tensile modulus, GPa	4.8	5.4	
BP	Cold	3	Buckling strength, MPa	1770	1700	53
E-glass				1800	1800	
BP			Buckling modulus, GPa	54	52	
E-glass				55	56	
BP	Cold	1.7	Tensile strength, MPa	1120	1206	55
			Tensile modulus, GPa	53.2	52.7	
BP	Moderately warm	2.5	Buckling strength, MPa	1209	1094	56
			Compression strength, GPa	410	427	
	Cold	2.3	Buckling strength, MPa	1209	780	
			Compression strength, GPa	410	428	

In [1], the mechanical properties of BP reinforced with finely cut roving, fabrics, or uniaxially oriented continuous BF are compared with carbon and fiberglasses based on unsaturated polyesters, epoxyphenol and phenol-formaldehyde matrices. It has been shown that over 12 months of climatic tests in the South Caucasus, a decrease in strength is observed, depending on the type of polymer matrix and the magnitude of the applied tensile stress. Aging processes have been shown to develop on the surface of the samples, but no experimental evidence of this statement has been given.

A comparison of the climatic resistance of BF and GF has been conducted by the accelerated method according to the Japanese standard JIS A1415 [24]. This standard provides for continuous irradiation of samples with a 60 Wt/m^2^ xenon lamp in the wavelength range of 300–400 nm at a temperature of 63 ^о^С and a relative humidity of 50% with irrigation (sprinkling) for 18 min every 2 h of exposure. For the given mode, 200 h of accelerated tests are equal to a year of climatic exposure. After 4000 h of testing (an analogue of 20 years of climatic exposure), the tensile strength of GF decreases from 2182 MPa to 1828 MPa (by 16%), then for BF a similar decrease is 13%, and the rate of the decrease of this indicator in BF is almost 2 times lower than that in GF.

The authors of [52] studied BP plates for construction purposes based on unidirectional fabric from BF, basalt roving, and DGEBA epoxy polymer. The samples have been tested in a climatic chamber in which, according to ISO 15686, a test mode is created simulating the climate of Italy: a 7-hour cycle combining dry atmosphere with a temperature of 60 °C and a relative humidity of 10%, a humid atmosphere with a temperature of 2 °C and a relative humidity of 80%, and stage ultraviolet radiation at a temperature of 35 °C and a relative humidity of 87%. To compare the results of accelerated tests, similar BP plates are exposed for 12 months under the natural conditions of Palermo.

At the initial stage of exposure, an increase in mechanical properties has been noted, as caused by the after-curing of the epoxy matrix. The methods of dynamic mechanical analysis and calorimetry have been applied to show conflicting results in composites and separately cured resin, which may be due to the uncontrolled impacts of moisture. According to alterations of the tensile strength and Young's modulus in bending, the equivalence of 56 days of accelerated testing is shown to be 1 year of climatic aging. It has been concluded that the studied BP is resistant to effects of a dry and humid climate, however, its mechanical parameters are unstable and fluctuate within 30–40%.

In [53], the mechanical properties of reinforcement with a diameter of 5 and 5.5 mm based on polyester and epoxy binder and unidirectional GF and BF after 3 years of storage in the cold climate of Yakutsk are compared. The methodical approach reviewed in [54] has been applied in measurements of the buckling strength and the buckling modulus. At a temperature of -60 °C, these indicators increase by 20%. During 3 years of testing at the warehouse, the BP and GP indicators remain at the initial level. The similar results have been obtained in [55]. Basalt-plastic reinforcement (BPR), which are unidirectional bars of a periodic profile with a diameter of 6–10 mm, manufactured according to standard TU 2296-001-86166796-2013 "Non-metallic composite reinforcement made of basalt plastic" are studied. Following 20 months of exposure in the climate of Yakutsk, an insignificant increase in the strength of the BPR have been observed.

A more detailed study of the climatic aging of these BP rods is given in [56]. Unidirectional rods of a periodic profile with a diameter of 6.8,10,16 and 20 mm based on an epoxy matrix have been exposed for 30 months on open stands in the moderately warm marine climate of Gelendzhik and 28 months on similar stands in the extremely cold climate of Yakutsk. For the initial and exposed BPR samples, insignificant changes in the mechanical parameters were determined. An increase in the compressive strength in Yakutsk by 4–12% was revealed. After exposure in Gelendzhik, this figure decreases by 10–17%. Using the methods of thermomechanical analysis and dynamic mechanical analysis, the shift of the $\alpha_{1}$-transition to lower temperatures, and the $\alpha_{2}$-transition to higher temperatures, was found. These effects are accompanied by an increase in the coefficient of linear thermal expansion, the diffusion coefficient of moisture, and the maximum moisture saturation after the climatic effect of BPR. Studies have shown high climatic resistance of BPR.

## 6 Conclusion

The analysis has shown that BF is a good alternative to GF for creating composite materials for various purposes. Even in the most aggressive alkaline environments, BP has a resistance comparable to or even higher than that of GP. If necessary, it is possible to increase their resistance to chemically active environments by regulating the composition of BF, applying protective layers, heat treatment of fibers, etc.

The comparison of resistance of BF, GF, and composite materials made on their basis to effects of temperature, moisture, solutions of salts, acids, alkalis, and exposure to natural environment was mostly performed under laboratory conditions applying accelerated methods. The obtained results provide us with a good basis for comparison of mechanical properties of new BP and their analogues, but they are not sufficient to obtain substantiated forecasts about the state of this class of materials under natural environmental conditions and under operation. In the majority of the research conducted, the duration of exposure to aggressive environments makes several months, and only in a few cases it is more than a year.

Thus, for expanded usage of BP as effective construction material under various climatic conditions, inclusive, that of the Arctic, it is reasonable and effective to perform long-term tests (a decade-long or more) of other composite materials tested before [41, 56, 57, 58], with monitoring of alterations in their mechanical properties and an analysis of developing processes of climatic ageing of BP [36, 37, 38, 39, 40, 41].

## Declarations

### Author contribution statement

All authors listed have significantly contributed to the development and the writing of this article.

### Funding statement

This work was supported by Project of the Russian Foundation For Basic Research (№18-29-05012) “Elaboration of Scientific Basis of New Composite Materials under Abiogenic and Biogenic Factors in the Arctic and Subarctic zones of the Sakha Republic (Yakutia)".

### Competing interest statement

The authors declare no conflict of interest.

### Additional information

No additional information is available for this paper
